# León (Spain) Health professionals’ knowledge and clinical practice towards the relationship between cardiovascular diseases and periodontal disease

**DOI:** 10.4317/jced.60529

**Published:** 2023-06-01

**Authors:** Juan-Manuel Gómez-González, Mariano del Canto-Díaz, Clara Jacobo-Orea, Miguel-Angel Alobera-Gracia, Mariano del Canto-Pingarrón

**Affiliations:** 1 DDS, Máster de Cirugía Bucal, Implantología y Periodoncia de la Universidad de León, España; 2 DDS. Profesor del Máster de Cirugía Bucal, Implantología y Periodoncia de la Universidad de León, España; 3 MD, DDS, PhD. Director del Máster de Cirugía Bucal, Implantología y Periodoncia de la Universidad de León, España

## Abstract

**Background:**

Cardiovascular pathologies have a high prevalence in the geriatric population, with acute myocardial infarction being one of the main causes of death in Spain. These pathologies have a systemic inflammatory component that is of vital importance. We also know in dentistry that the main gingival pathogens are capable of generating a systemic inflammatory response, being indirectly involved in the development of the atherosclerotic lesion, assuming, therefore, that periodontal disease is a cardiovascular risk factor. The objective of this study is to determine the knowledge of health professionals who treat cardiovascular diseases about periodontal disease and its relationship with heart disease.

**Material and Methods:**

A health survey was carried out on 100 Cardiologists, Internists and General Practitioners in the province of León. Points of interest in this survey: the professional’s own oral health, knowledge of the relationship between periodontal and heart disease and, lastly, the training received in medicine on oral health.

**Results:**

60% of professionals reviewed their oral health annually and 20% randomly. 48% of health professionals were unaware of periodontal diseases, 77% claimed to have not received university training in this regard, only 13% of those surveyed acknowledged having received more than 10 hours of training on oral health in their experience and finally, 90% thought that training in both Medicine and Dentistry should be collaborative.

**Conclusions:**

The degree of knowledge of health professionals regarding oral health is poor (77%), therefore the number of collaborative consultations with dental professionals is low (<63%). Training projects targeting a correct preventive medicine are shown to be necessary.

** Key words:**Cardiovascular disease, oral-systemic health, periodontitis, knowledge, physicians.

## Introduction

Cardiovascular diseases are highly prevalent in the Spanish population, the most frequent being heart failure and ischemic heart disease. Acute myocardial infarction is one of the leading causes of death in the country (1). All these pathologies have a common systemic inflammatory component whose knowledge is of vital importance to understand them. The fact that periodontal disease is one of the most common human diseases (2) and coronary heart disease the most important cause of death in developed countries is the reason for the great interest aroused in both the dental and medical scientific communities to find a possible relationship between both entities.

Today it is known that the main pathogens originating from the subgingival biofilm (*Aggregatibacter actinomycetemcomitans*, *Porphyromonas gingivalis* and *Tannerella forsythia*) can generate a systemic inflammatory response (C-reactive protein, homocysteine, plasmatic fibrinogen, factor VII and lipoproteins), being involved in the development of atherosclerotic lesion as a new route of indirect action (3).

Therefore, it can be assumed that periodontal disease is a cardiovascular risk factor, so primary care physicians, cardiologists, and dentists must have sufficient knowledge about this highly relevant issue in public health.

In 2014, the Spanish Societies of Periodontics and Cardiology founded a working group that published a SEC-SEPA consensus document in 2016. The “Manifiesto Periodoncia y Salud General” is a call to the oral-health community and health professionals with the objective of promoting the prevention and early detection of periodontal diseases and their treatment in order to avoid their consequences on general health. The statement includes the questionnaire “Cuida tus encías”, with the aim of helping General Practitioners and Cardiologists to identify the first signs of periodontal pathology and thus be able to prevent it (4).

Objectives

The present study is based on the following hypothesis: the prevention of periodontal disease may be of importance for the prevention of cardiovascular diseases given their relationship.

The objectives set for this study are the following:

1. To determine the degree of knowledge that health professionals who treat heart disease in the health area of León have about oral-periodontal health.

2. To determine the opinion of these professionals on the interrelationship of cardiovascular diseases and periodontal disease.

3. To study the collaborative consultations that are carried out between doctors and dentists.

## Material and Methods

1) Bibliographic research

Bibliographic research was carried out in the main scientific databases: Pubmed through the Medline search engine, Cochrane, Google Scholar and Scopus. The key terms used were the following: Cardiovascular disease, Dental education, Interdisciplinary education, Oral-systemic health, Periodontitis.

The search was limited to studies published in the last 10 years in Spanish and English. Those selected publications were requested to study a particular population of health professionals that usually worked with patients that suffered from cardiovascular diseases. The method of those studies had to be through a survey or an interview. A total of 8 scientific articles that met the established inclusion criteria were obtained.

2) Survey selection

The several surveys included in the different studies were evaluated. The aim was to later carry out a translation of one of them and use it as a tool in the selected population of the present study. Selection criteria were established. The survey of the publication had to be easily reproducible and adaptable to the target sample (in this case the health professionals from the health area of León), the study had to have been carried out on a specific population of professionals and not generalized for a better comparison of results. Finally, the study had to contain a series of variables to be questioned in accordance with the objectives presented:

- Knowledge of professionals about oral health.

- Knowledge of professionals about the relationship of periodontal and cardiovascular diseases.

- Professional practice and collaborative consultations with dentists and stomatologists

The study selected for the translation and application of the survey was: “Megan Mosley *et al*. “North Carolina Cardiologists’ Knowledge, Opinions and Practice Behaviors Regarding the Relationship between Periodontal Disease and Cardiovascular Disease” (5).

3) Survey translation and creation

Since the original version of the questionnaire is written in English and adapted for a North Carolina population, translation and cultural adaptation to Spanish and Spain (León) was necessary. For this, the rules established by Beaton & Bombardier were followed. It involved the use of a tool in another country and with a different language, so its suitability must be based on both a correct translation and an adequate cultural adaptation (6).

Translation and synthesis: Two people qualified in English (English speakers or qualified with a C1 level) intervened in this first step of adaptation and translation. These people were in charge, separately and without communication between them, of carrying out a translation of the questionnaire into Spanish, obtaining two new questionnaires T1 and T2. In this step, an adaptation to the culture and target population of the study was also carried out. Specifically, in countries with different healthcare systems (United States, a privatized system and Spain, a public system) the questions were reoriented to make more sense in the sample studied. Subsequently, with the two questionnaires T1 and T2 already written, the author of the study elaborated a synthesis of both in a third final questionnaire T12, which would be the object of study in the following steps so that to be validated as a correct translation and, therefore, as a survey to be used for the study.

Reverse translation: Two other different people with the same qualifications in English than those of the initial translation were asked to retranslate the T12 synthesized questionnaire into the original language. As in the first step, the translation was done separately. Thus, two new questionnaires in the original language BT1 and BT2 were obtained.

Resolution: The author of the study gathered the results obtained from the translation process. The discrepancies obtained between the BT1 and BT2 surveys were evaluated. In addition, the opinion provided by the translators regarding the translation process and its complexity was considered. It was decided that the final translation of the original T12 survey was correct and therefore valid to study on a sample similar to the one published by Megan Mosley *et al*.

The survey was exported into digital format using the Google Forms system. It was entitled: “Enfermedades Cardiovasculares y Salud Periodontal. Estudio realizado a profesionales de la Medicina en el Área de Salud de León”.

The following inclusion criteria were applied to the studied population:

- Medical professionals who treated heart diseases (Cardiologists, General Practitioners and Internists).

- Professionals attending the health area of León.

- Professionals from both the public and private health sectors.

## Results

The results obtained in the surveys were divided into 5 blocks according to the thematic of the question:

1. Professional profile.

2. Oral health of the professional.

3. Collaboration with dental professionals.

4. Opinion on the relationship that periodontal diseases have with systemic health.

5. Training on oral health.

1)Professional profile

The survey was distributed on a total of N=100 health professionals. The profiles of these professionals were diverse. 100% of those surveyed carried out their professional practice within the public sector of health, 7% also combined this practice in the private sector. The professionals surveyed belonged to the specialties of: Cardiology, Internal Medicine, and General Medicine.

**Figure 1:**
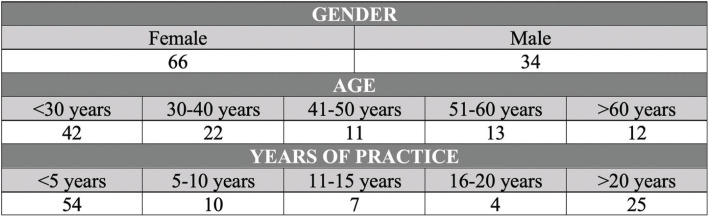
Descriptive results about the population of the study. Professional profiles. N=100.

The ratio in terms of sex turned out to be 2 to 1 in favor of the female gender. The profile of age and years of experience was diverse. The sample included residents with less than 5 years of experience and associate professionals with more than 20 years of experience in their respective services (Fig. 1).

2) Oral health of the professional

One of the blocks of questions in the survey dealt with the oral health of the professionals and their perception of it. These questions were intended to value the importance they gave to oral health. Since the oral health of the professional themselves could reflect the importance that they gave to the oral health of their patients.

**Figure 2:**
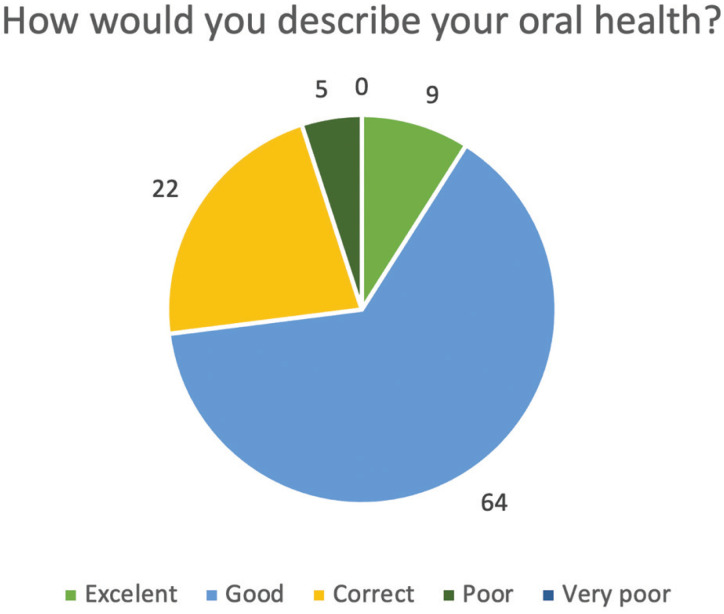
Answers to the question: “How would you describe your oral health?”.

61% of those surveyed arranged oral examinations with a frequency of less than one year, while of the remaining 39%, 17% did them biannually and 21% acknowledged that they did not arrange it periodically. Only one of the respondents acknowledged never having had an oral examination done. On the personal perception of the professionals: 9% would rate their oral health as “excellent”, 64% as “good”, 22% as “correct” and 5% as “poor”. There was, therefore, a discrepancy regarding the perception and the periodic objective examinations carried out on the respondents. 33% of those surveyed admitted having ever been diagnosed with Gingivitis or Periodontitis (Fig. 2).

3) Collaboration with dental professionals

When asked about the consultations made with professionals in the field of dentistry, it was observed that only 42% of the respondents referred their patients to the dentist for the pertinent periodontal assessment combined with their cardiological treatment. Similarly, only 13% reported having received consultation from more than 6 patients reciprocally.

Today we know that periodontal pathology turns out to be one of the most prevalent in the population, the sixth most prevalent pathology. We know that 1 in 3 Spanish adults have periodontal attachment loss greater than 3mm. Associating this with the survey, we can deduce that the degree of referrals is very low (7).

4) Opinion on the relationship that periodontal diseases have with systemic health

**Figure 3:**
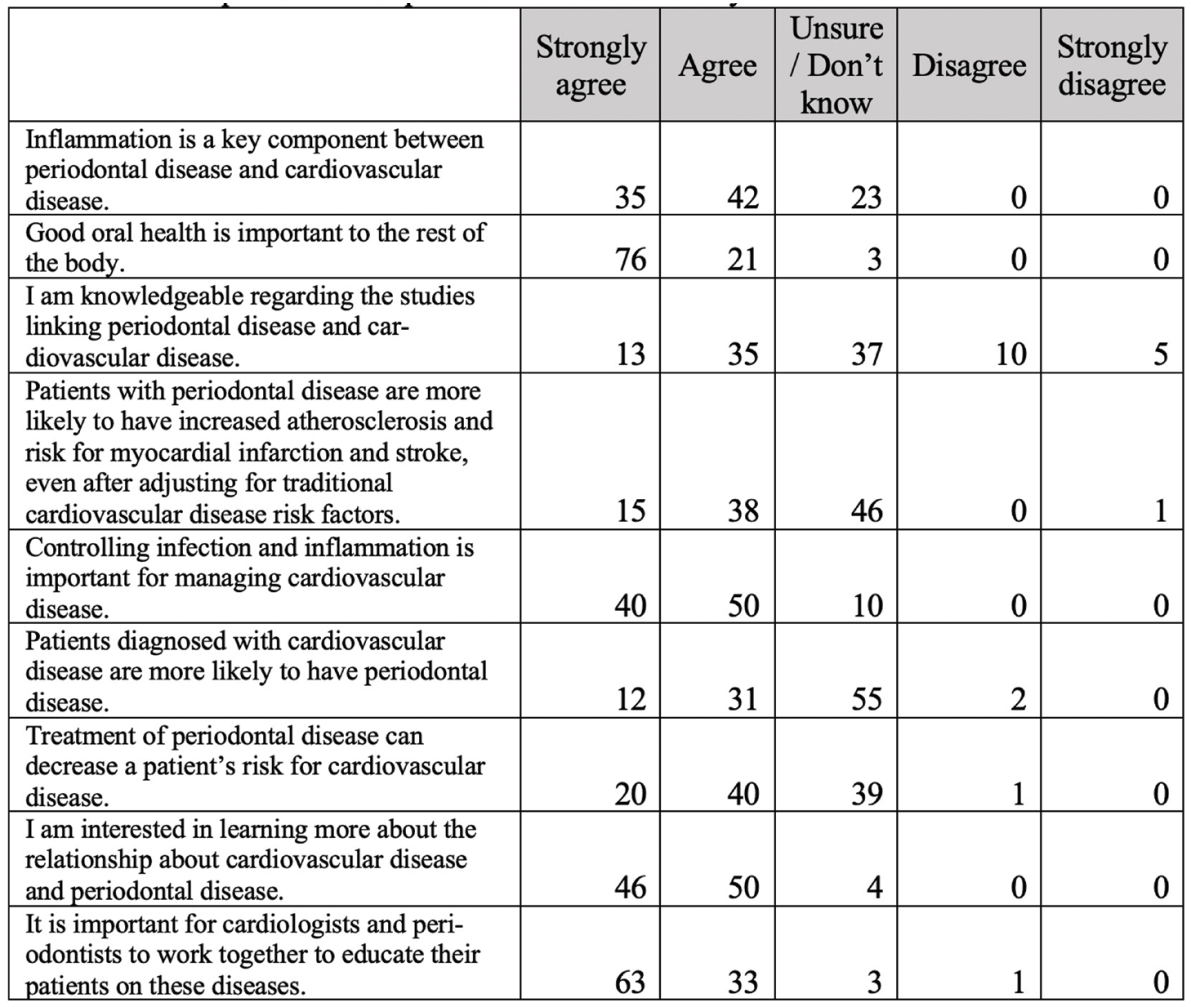
Opinios about periodontal disease and systemic health.

Knowledge about inflammation at the systemic level as a key to understanding cardiovascular diseases was widespread (77%), as well as that correct oral health is relevant for adequate systemic health (97%). Around half of those surveyed (52%) admitted not having information or knowledge about the studies that interrelate cardiovascular and periodontal pathologies. 47% were unaware that patients suffering from periodontitis have a greater risk of suffering atherosclerosis and that, conversely, patients with heart disease could have a greater chance of developing periodontal pathology (55%). In general, half of the sample studied was unaware of the interrelationships of these pathologies and the efficacy of applying a correct preventive medicine acting on these interrelation pathways. However, it was believed that medicine and dentistry should work more collaboratively (96%) and there was general interest in receiving more information on the subject (96%) (Fig. 3).

5) Training on oral health

In order to understand this widespread unawareness on the subject, the last block of questions in the survey attempted to determine the training received by the surveyed professionals on oral health throughout their years of experience.

62% of those surveyed stated that they had not received any type of training on oral health in their years of experience, 38% of those surveyed stated that they had not received any type of information throughout the 6 years of their degree and had only completed hours of professional training later in their corresponding jobs. Only 13% of those surveyed acknowledged having received a total of more than 10 hours of training on oral health in their professional career.

**Figure 4:**
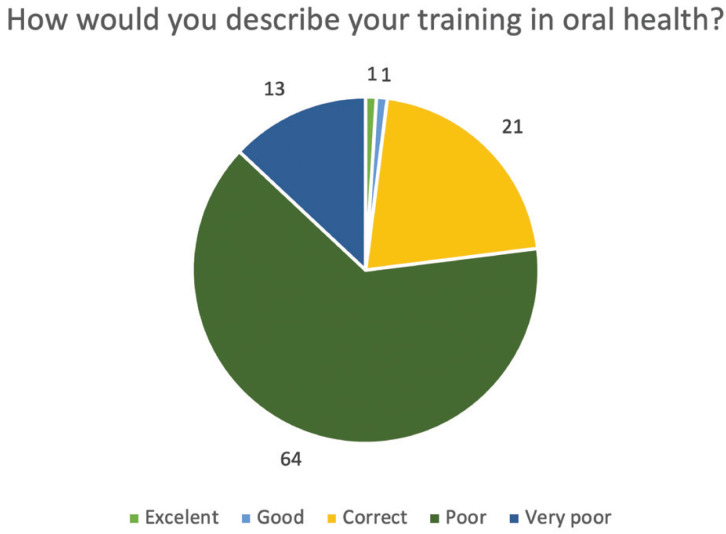
Answers to the question: “How would you describe your training in oral health?.

To the question, How would you rate your training in oral health? Only 21% would rate it as correct to carry out their job, while 77% would rate it as poor or very poor. In contrast to the results obtained on training, 64% of those surveyed claimed that in their respective jobs they were required to be able to perform a basic oral examination correctly (Fig. 4).

## Discussion

So that the results obtained can be extrapolated to the population of health professionals in general, they must be compared with similar studies aimed at different samples in other locations. The results were compared with the 8 scientific articles filtered after the initial bibliographic search of the study. The geographical diversity that can be observed and the cumulative sample of professionals surveyed allowed to conclude and deduce with statistical relevance.

- Mosley M *et al*. → North Carolina (United States) (5).

- Betul Aycan A *et al*. → Stambul (Turquey) (8).

- Arya V *et al*. → Mangalore (India) (9).

- Alexia V *et al*. → *Pi*rineys (France South) (10).

- Tasdemir Z *et al*. → Kayseri (Turquey) (11).

- Dubar M *et al*. → Lille (France North) (12).

- Asa′ad F *et al*. → Riyadh (Saudi Arabia) (13).

- Kashefimehr A *et al*. → Tabriz (Iran) (14).

When comparing the results obtained with the article published in North Carolina on which the survey was based, similar results could be observed. The Mosley *et al*. sample (N=119) was predominantly male and belongs to the private sector of Cardiology. However, it was agreed with this study that approximately 50% of those surveyed were unaware or did not believe that a correct diagnosis and periodontal treatment can be a cardioprotective factor. Only 18% knew about the etiology of periodontal disease and could perform a basic oral examination. In the same way, it was agreed that doctors and dentists should receive training focused on collaboration and that there is interest in the subject among professionals in the sector (5).

In the study published in Mangalore (India) by Arya *et al*., more positive knowledge results were observed. Of the total sample (56), 87.5% took in consideration the signs of periodontal pathology they usually explored (in this case bleeding and crown lengthening) as cardiovascular risk factors. However, only 40% acknowledged asking their patients questions about oral health in their routine consultations (9).

Nuances were observed in the studies published in Turkey. Betul Aycan *et al*. oriented their survey around endodontic-periapical pathology. Endodontic pathology was described as a conspicuous, serious infection with great cardiology interrelation and dissemination, 66% of cardiologists and 80.3% of cardiac surgeons claimed to refer their patients to the dentist in case of any suspicion of odontogenic infection and placed great importance on prophylaxis against bacterial endocarditis. However, no reference was made to Periodontitis in this study (8). The study by Tesdemir *et al*. introduced general systemic health related to Periodontitis into the equation: there was general knowledge of the influence of Periodontitis on systemic health (90.8%). The most related systemic pathology was Diabetes Mellitus (66.8%), no reference was made to cardiovascular diseases (11).

In Saudi Arabia, Riyadh *et al*. described results along the same lines. Their sample was predominantly resident professionals in training, so their knowledge could be seen as more recent. 49.8% never asked questions about oral health in consultation, 93.2% claimed not to have received any type of training on oral health and 23.5% described the fact of performing a basic periodontal examination as with lack of security and discomforTable. 33% had never referred any patient to the dentist (13).

Similar results were also described in Iran. The sample of Kashefimehr *et al*. (N=54 cardiologists) allowed to obtain the following conclusions: 82% observed inflammation as a common component between periodontitis and cardiovascular diseases and 72% understood that the control of this infection and inflammation may have an important role in cardiovascular prevention. However, 62% had not received training on oral health and 80% believed that doctors and dentists should be trained collaboratively (14).

Finally, in France, Alexia *et al*. described a sample (N=222) with general notions about the interrelationship of said pathologies. Unawareness was described towards other related pathologies such as obesity, respiratory pathology and joint pathology. More than half of their sample claimed to be concerned about the oral health of their patients, but 94% believed that this consideration of periodontal disease was not enough (10). In the other French study by Dubar *et al*., similar results to those published were described. in Turkey, the relationship of Diabetes to Periodontitis was given more importance (75%) than cardiovascular disease (53%). Few of the professionals (<30%) associated Periodontitis with other pathologies such as Rheumatoid Arthritis and Alzheimer’s. 74% acknowledged that they did not ask their patients questions about oral health or perform basic oral examinations. 86% admitted that their knowledge on the subject was not enough (12).

In general, similar results were observed in the different geographical points in which surveys among health professionals had been carried out. It can be deduced that professionals acknowledge that inflammation is key to understanding cardiovascular diseases, that this inflammation can come indirectly from periodontal diseases and that acting at this level can imply scientifically based prevention. However, in daily clinical practice, this prevention is not being carried out effectively. The interest shown by professionals and the widespread lack of collaboration with dentistry make evident the need to create a more effective, protocolized and standardized preventive action plan.

The unanimity in the global results does not make it necessary to carry out studies similar to this one, since it demonstrates the need for a change in the form of action towards health at a global level, prioritizing prevention over treatment.

## Conclusions

In view of the objectives set out in the study, it can be concluded that:

1. The degree of knowledge of health professionals regarding oral health is poor (77%), most claim not to have received university training in this regard (62%). The hours of subsequent professional training do not exceed 10 hours (87%).

2. Notions about the influence of periodontal disease on cardiovascular diseases are diverse, 50% claim not to know this relationship, but there is unanimity in the interest in learning more about the matter.

3. The number of collaborative consultations with dentists is low <63%. It is believed that doctors and dentists should work collaboratively (99%).

Today, the influence of periodontal pathologies on systemic health in general is well known and proven. However, given this premise, in daily clinical practice it is not depicted that correct preventive medicine is being carried out. Training projects targeting health professionals are shown to be necessary, since cardiovascular diseases represent a great issue for public health. A correct health education should be carried out: there is no better treatment than prevention.
